# Effects of ploidy variation on promoter DNA methylation and gene expression in rice (*Oryza sativa* L.)

**DOI:** 10.1186/s12870-018-1553-5

**Published:** 2018-11-29

**Authors:** Hongyu Zhang, Asif Ali, Feixue Hou, Tingkai Wu, Daiming Guo, Xiufeng Zeng, Fangfang Wang, Huixia Zhao, Xiaoqiong Chen, Peizhou Xu, Xianjun Wu

**Affiliations:** 211-Key Laboratory of Crop Genetic Resources and Genetic Improvement, Ministry of Education, Institute of Rice Research, Sichuan Agricultural University, Huimin Road, Chengdu, 611130 China

**Keywords:** WGD, DNA methylation, mRNA expression, Digital gene expression, MeDIP sequencing, Sodium bisulfite sequencing

## Abstract

**Background:**

Polyploidy, or whole-genome duplication (WGD) promotes genetic diversification in plants. However, whether WGD is accompanied by epigenetic regulation especially DNA methylation remains yet elusive. Methylation of different region in genomic DNA play discrete role in gene regulation and developmental processes in plants.

**Results:**

In our study, we used an apomictic rice line (SARII-628) that produces twin seedlings of different ploidy for methylated DNA immunoprecipitation sequencing (MeDIP-seq). We compared the level of methylation and mRNA expression in three different (CG, CHG, and CHH) sequence contexts of promoter region among haploid (1X), diploid (2X), and triploid (3X) seedling. We used MeDIP-Seq analysis of 14 genes to investigate whole genome DNA methylation and found that relative level of DNA methylation across different ploidy was in following order e.g. diploid > triploid > haploid. GO functional classification of differentially methylated genes into 9 comparisons group of promoter, intergenic and intragenic region discovered, these genes were mostly enriched for cellular component, molecular function, and biological process. By the comparison of methylome data, digital gene expression (DGE), mRNA expression profile, and Q-PCR findings LOC_ Os07g31450 and LOC_ Os01g59320 were analyzed for BS-Seq (Bisulphite sequencing).

**Conclusions:**

We found that (1) The level of the promoter DNA methylation is negatively correlated with gene expression within each ploidy level. (2) Among all ploidy levels, CG sequence context had highest methylation frequency, and demonstrated that the high CG methylation did reduce gene expression change suggesting that DNA methylation exert repressive function and ensure genome stability during WGD. (3) Alteration in ploidy (from diploid to haploid, or diploid to triploid) reveals supreme changes in methylation frequency of CHH sequence context. Our finding will contribute an understanding towards lower stability of CHH sequence context and educate the effect of promoter region methylation during change in ploidy state in rice.

**Electronic supplementary material:**

The online version of this article (10.1186/s12870-018-1553-5) contains supplementary material, which is available to authorized users.

## Background

Methylated cytosine (also known as fifth nucleotide) is one of known epigenetic mark that is extensively found in genome of eukaryotes [1, 2]. Methylated cytosine (mCs) plays an important role in gene regulation in order to control growth and developmental processes in plants [3]. Methylation of genome affects morphology, stability, differentiation, regulation of gene expression, transposable elements (TEs) transposition, chromatin structural stability, and protection of genome from invasion [4–8]. In general, DNA methylation can be stably inherited to trans-generations by mitotic cell division and considered as a heritable epigenetic mark in plant [9]. Methylated cytosine in plants comprised of three sequence contexts: symmetric (CG and CHG) and asymmetric (CHH) methylation depending on the composition of base (where, H refers to A/T/C). Methylation at CG sequence context is the major type of cytosine methylation, as it is widely distributed not only in heterochromatic (such as TEs and repeat sequences) but also in eu-chromatic regions of genes [10, 11]. Methylation is believed to be primarily catalyzed by a specific families of DNA METHYLTRANSFERASES; MET1 (homologous to animal DNMT1) for CG, plant-specific CHROMOMETHYLTRANSFERASE (CMT3 and CMT2) for CHG, and DRM2 (homologous to animal DNMT3) for CHH sequence context. CMT2 is also found to be involved in the maintenance of DNA methylation in CHH sequence context [12–19].

BS-seq (Bisulfite sequencing) is a gold-standard method to study DNA methylation, which can provide a genome-wide methylome analysis at single-base resolution in model plants [20, 21]. Xu et al. [22] used BS-seq to measure DNA methylation in castor bean seeds. They found CHH methylation sequence context was substantially higher in endosperm and embryo than previously known tissues of plants. Compared with embryo, the endosperm exhibited a significant reduction in CG and CHG sequence contexts and non significant reduction in CHH sequence context methylation. Feng et al [1]. compared DNA methylation patterns in eight diverse species of plants and animals, and established TEs and repeat sequences revealed a high degree of methylation, with the highest and lowest degree of methylation in CG and CHH sequence context, respectively. Genomic DNA mostly covers CG sequence context methylation, that emphases on exons rather than introns. CHH sequence context methylation is more widely distributed in monocot genome (e.g. rice) possibly due to massive distribution of repeated sequences. Lee et al. [23] found that repeated sequences and TEs had high levels of CHG sequence context methylation and implied role of DNA methylation in evolution. Hisataka et al. [24] showed that there are many TEs and repeated sequences in rice genome and changes in CG and CHG sequence context methylation were not directly associated to gene function in rice.

Polyploidy or WGD is fundamental state of plants to ensure diversification under unfavorable environments [25, 26]. Following genome doubling, a specie undergoes a process of “diploidization” and evolves into new a contemporary diploid specie. Although, a large number of repeated genes are lost during diploidization, but still plant genome contains a large fraction of repeated genes; for example, these repeated sequences account for 25% of the genes in Arabidopsis [27], 30% in poplar [28], 50% in rice [29], and 67% in soybean [30]. Li et al. [31] recently graphed the fate of repeated genes in nearly 40 different flowering species and found that most of the genes that had undergone one or more WGD events were quickly restored back to single copy state. However, yet some of genes were remained duplicated. Most of those genes belong to gene family that produces accustomed responses to biotic and abiotic stresses. In rice, changes in the patterns and levels of gene methylation caused expression differences. These differences depend on the mode of duplication; the direction (positive or negative) of correlation and K_s_ (synonymous amino acid substitution rate) of WGD vs. single gene duplication [32]. In soybean, WGD of genes that have more methylation in CG sequence context showed higher expression levels and were more likely to be retained as repeats [33]. In cassava, a strong positive correlation between DNA methylation and expression of repeated genes was found during WGD [34].

Previous studies have revealed that occurrence of many polyploids was caused due to external stimuli, such as pressure and environmental fluctuations or the type of reproductive system that ultimately lead to the formation of un-reduced gametes [35–38]. Polyploids can overcome the fluctuations, as their genomic background is more malleable; therefore, polyploids have better potential and rapid adaption under diverse environment than that of diploids [39, 40]. Although, in a stable environment, the extinction risk for polyploids is higher than that of diploids; thus, only challenging environments can increase environmental stability and specific adaptability of polyploids by chromosome rearrangement and thereby reduces the risk of extinction [41, 42]. Kagale et al. [43] showed that at-least eight WGDs occurred in Cruciferae species that corresponds to five independent polyploidization events. This correlation between WGD and diversification of species demonstrated that environment played an important role in occurrence of WGD.

Earlier studies have focused in the variation of DNA methylation caused by polyploidization of allopolyploids [44–48], while autopolyploid methylome analysis also clearly distinguished the epigenetic variation in TEs and gene expression induced by genomic doubling [49]. In current study, we used an autopolyploid spontaneously occurring apomictic line SARII-628. It often produces twin seedlings of different ploidy including monoploid, diploid and triploid [50–53]. Methylome analysis of SARII-628 (1X:2X:3X) showed that DNA methylation patterns were found different among different ploidy levels [50]. Although, it was still unknown whether the methylation patterns in CG, CHG, CHH sequence context of promoter regions are affected by different ploidy level or not. Therefore, it is of great importance to study (1) methylation patterns of promoter e.g. intergenic and intragenic regions and further analysis the plant buffering method [54] among different ploidy levels. (2) discover the effects of different ploidy in CG, CHG, CHH sequence context methylation.

## Results

### Confirmation of ploidy of haploid, diploid, and triploid plants from twin seedlings

**Fig. 1:**
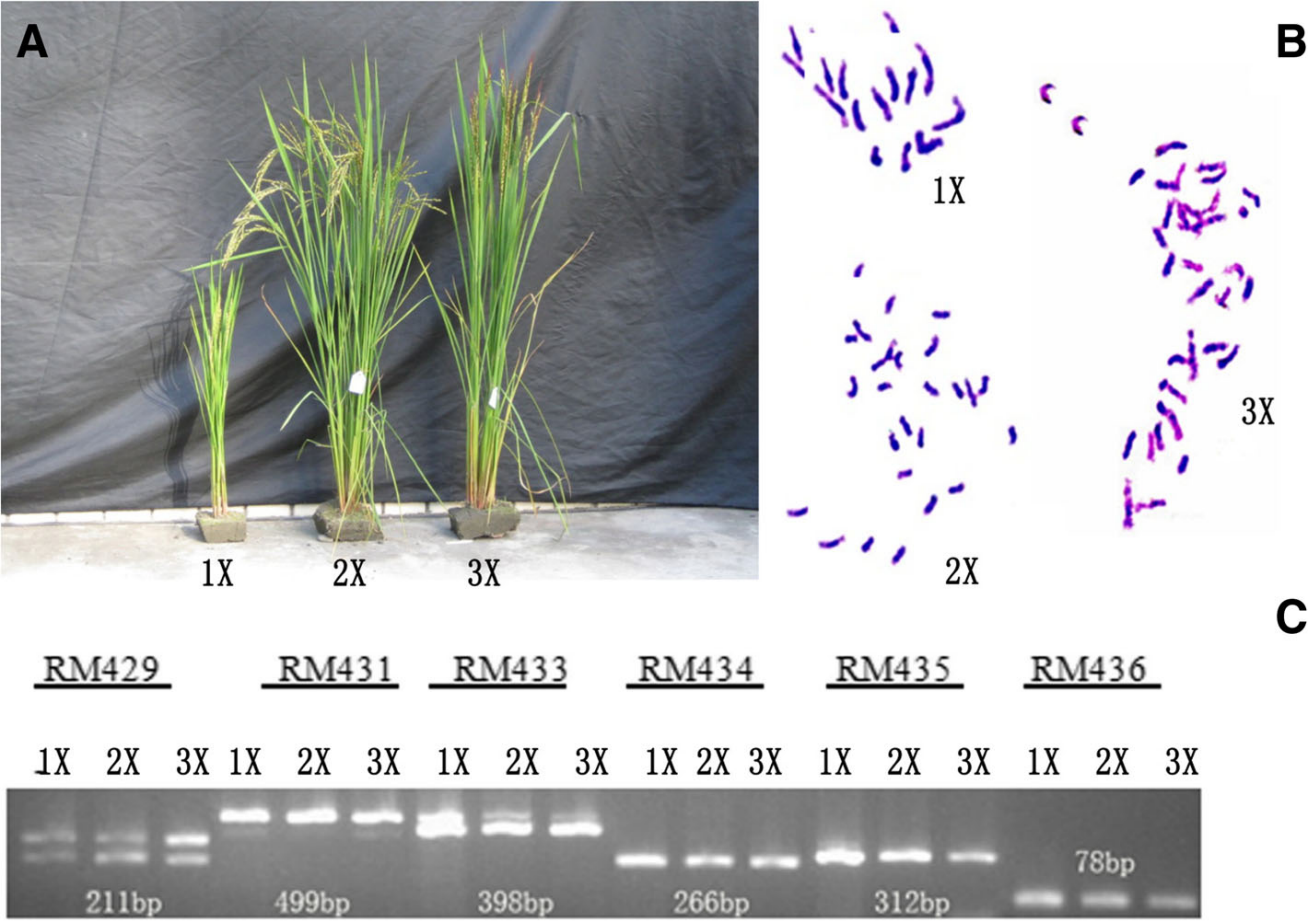
**a** Phenotypic comparison of haploid, diploid, and triploid rice plants. 1X, 2X and 3X are representing haploid, diploid and triploid plants respectively**. b** Chromosome counts of root tip among different ploidy level. 1X, 2X and 3X are representing total chromosome counts in haploid, diploid and triploid plants respectively**. c** Agarose gel electrophoresis screening for six STR markers in in haploid, diploid and triploid plants. RM429, RM431, RM433, RM434, RM435 and RM436 are representing PCR product of 211, 499, 398, 266, 312, and 78 bp respectively

We observed significant morphological differences among haploid (1X), diploid (2X), and triploid (3X) plants that were grown under the same conditions (Fig. 1a). The haploid plants displayed reduced plant height, decreased numbers of tillers, short narrow leaves, and infertile spikelets. While, triploids were taller, showed increased numbers of tillers, leaf size, enlarged stem size, grain size, awn size, and infertile pollen due the different ploidy level than diploid. Root tip meristematic cells were used to count the chromosome numbers of each sample of different ploidy levels. The chromosome numbers of the putative 1X, 2X, and 3X seedlings were x = 12, 2x = 24, 3x = 36 found, respectively (Fig. 1b). These results showed that majority of differences observed in plant phenotypes were associated with ploidy levels.

### Screening of different ploidy with short tandem repeat (STR) markers

The polyploids from twin seedlings of SARII-628 are spontaneously occurring. Previous studies had shown that ploidy changes did not change the genome sequences [51–53]. We performed PCR amplifications in plants of different ploidy using 38 pairs of STR primers in order to ensure that there were no obvious genomic differences in the following amplification process. Results revealed that 38 primer pairs did not show the amplification of any extra bands in any of seedling of 1X, 2X, and 3X. These results have specified that genome of 1X, 2X, and 3X plants have not experienced major rearrangements. Figure 1c is showing the amplification of six STR primer pairs (RM429, RM431, RM433, RM434, RM435, and RM436).

### Detection of single nucleotide polymorphism (SNP) among different ploidy material

**Fig. 2:**
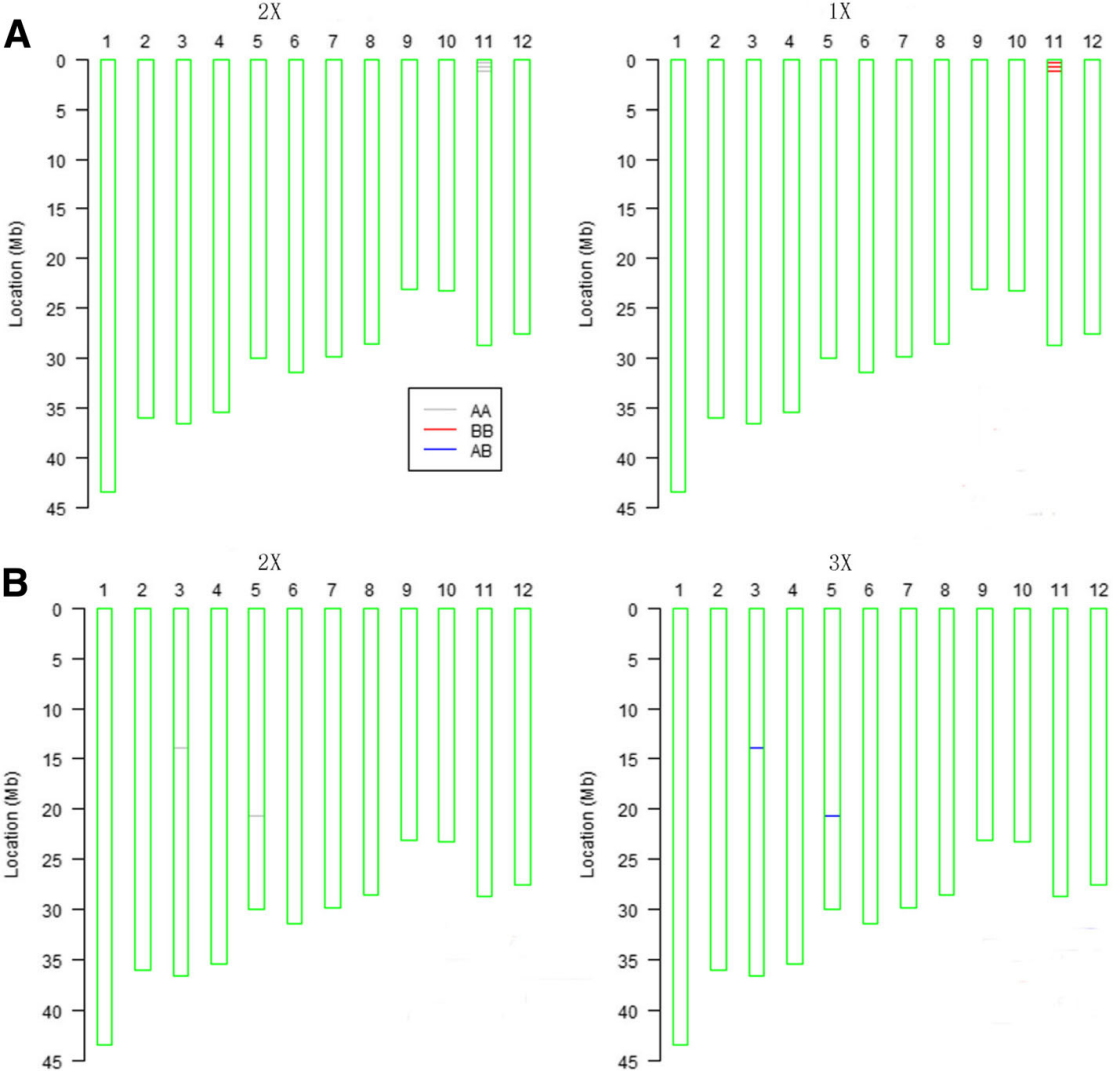
SNP array of haploid, diploid and triploid plants. **a** SNP comparison of diploid and haploid **b** SNP comparison of diploid and triploid. The short line represents a single nucleotide polymorphism at respective position, and the blank space represents absence of polymorphism at respective position. AA and BB represent pure-fit points, while AB represents a hybrid site

In order to determine the consistency of DNA sequences among haploid, diploid and triploid ploidy seedlings. We tested 6000 SNP variations at single sites in different ploidy DNA through a 6 K chip. SNP array showed DNA sequences of different ploidy levels had similar height, except a variance of 6 single bp loci (Fig. 2). Comparison of 1X and 2X revealed SNPs of four sites at 291791, 335364, 753,255 and 1,151,760 bp on 11th chromosome (Fig. 2a). In both diploid and haploid SNPs are of pure-fit sites. On the other hand, comparison of 2X with 3X revealed SNPs of 13,899,939 and 20,701,693 bp on 5th and 3rd chromosome respectively (Fig. 2b). The SNPs in the 2X are pure-fit sites and while in the 3X are hybrid sites.

### Gene body DNA methylation in different ploidy

**Fig. 3:**
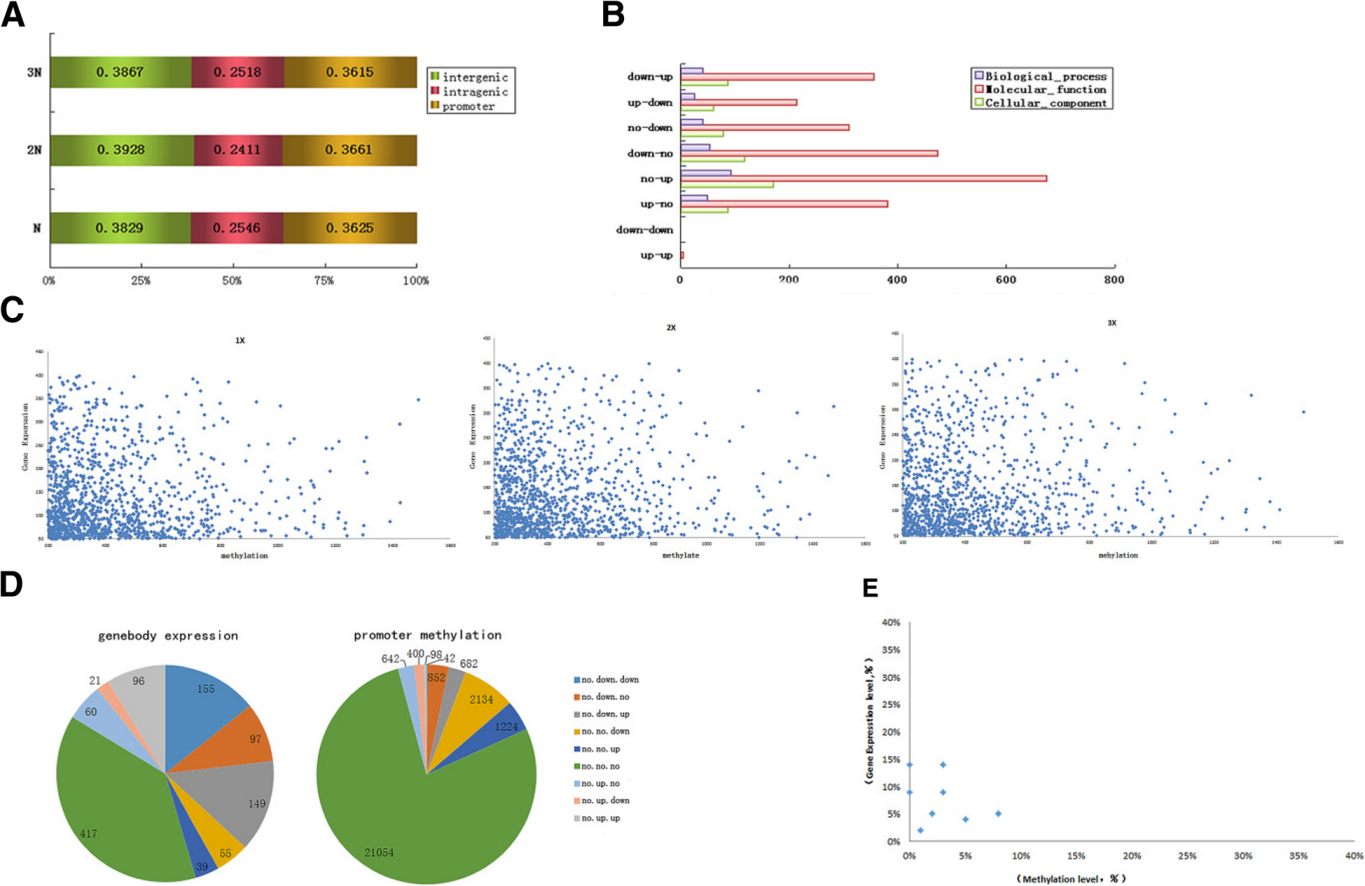
**a** Overall distribution of sequencing reads of DNA methylation in three different gene regions of 1X, 2X, and 3X. Green, red and yellow boxes are representing intergenic, intragenic and promoter region respectively. **b** GO functional classification of differentially expressed methylated genes in promoter regions of haploid, diploid, and triploid seedlings. Purple, red and green bars are representing categories of biological process, molecular function, and cellular components respectively. **c** The negative correlation between promoter DNA methylation and gene expression of 1X, 2X, and 3X **d** Analyses of relative gene expression and promoter region methylation levels in nine comparison groups for haploid, diploid, and triploid rice seedlings. Left and right pie graph are representing gene body expression and promoter methylation respectively. **e** The relationship between gene expression and promoter DNA methylation levels in nine comparison groups

MeDIP-seq [55] was used to analyze DNA methylation in different genomic regions of haploid, diploid, and triploid seedlings. In total, all three samples generated 498 million Illumina sequencing reads, and the number of unique reads in a single sample was > 50%. We determined the DNA methylation status in intergenic, intragenic and promoter regions (2 kb upstream of each gene) by calculating the percentage of methylated fragments mapped to each region. We found similar patterns of methylation distribution among all ploidy levels (Fig. 3a) with an average of 38.75% mapped reads located in the intergenic, 36.34% in the promoter, and 24.92% in the intragenic regions, indicating that DNA methylation occurs more frequently in intergenic followed by promoter and intragenic regions. Transposons are mainly distributed in the intergenic and promoter regions, and they are relatively more likely to be methylated than functional genic regions.

### Differential DNA methylation among 1X, 2X, and 3X seedlings in different gene regions

In order to understand whether the change in ploidy led to the relative changes in DNA methylation levels in different genomic regions, we compared the number of mapped reads in a pairwise manner between two ploidy levels in each region. Table 1 shows the nine comparison groups based on mapped reads being increased (up), decreased (down), or of no change (no). The cut-off of > 1.5-fold change of mapped reads in a designated region between two ploidy levels and *p*-values < 0.01; were used to determine the differences of methylation in each comparison (1X vs. 2X or 2X vs. 3X). If methylated reads were present in any of genomic regions but not in the corresponding region in other ploidy levels, this situation was also recognized as a significant change.

**Table 1:** Number of genes showing differentially methylated regions (DMR) for nine group comparisons in 1X, 2X, and 3X seedlings

DNA methylation levels	Promoter regions	Intergenic regions	Gene body regions
1X(no)- 2X(no)-3X(no)	29,095 (76.39%)	22,881 (72.69%)	33,657 (71.71%)
1X (no)- 2X no)- 3X (up)	2708 (6.86%)	2176 (6.91%)	2847 (6.07%)
1X (no)- 2X (down)- 3X (no)	1968 (4.98%)	1776 (5.64%)	2538 (5.41%)
1X (no)- 2X (up)- 3X (no)	1680 (4.25%)	1293 (4.11%)	2133 (4.54%)
1X (no)- 2X (no)- 3X (down)	1576 (3.99%)	1267 (4.02%)	2453 (5.23%)
1X (no)- 2X (down)- 3X (up)	1392 (3.53%)	1225 (3.89%)	1789 (3.81%)
1X (no)- 2X (up)- 3X (down)	1041 (2.64%)	838 (2.66%)	1455 (3.10%)
1X (no)- 2X (up)- 3X (up)	16 (0.04%)	16 (0.05%)	26 (0.06%)
1X (no)- 2X (down)- 3X (down)	8 (0.02%)	7 (0.02%)	39 (0.08%)
Total number	39,484	31,479	46,937

DNA methylation levels in three genomic regions responded to ploidy change similarly (Table 1). Group [1X (no)- 2X(no)- 3X(no)] accounted for 71.71 to 76.39% for different genomic regions, suggesting that DNA methylation is relatively stable up on ploidy level change. The analysis of other eight groups of methylation showed that (1X(no)- 2X(no)- 3X(up) and 1X(no)- 2X(down)- 3X(no) are the most likely patterns of methylation change in all three genome regions. Whereas, [1X(no)- 2X(up)- 3X(up)] and [1X(no)- 2X(down)- 3X(down)] are the least likely patterns of methylation change, suggesting ploidy increase does not always increase or decrease methylation based on ploidy level.

### GO enrichment analysis of differentially methylated genes

Gene Ontology (GO) enrichment analysis were conducted to genes based on genes showing differential DNA methylation in the eight comparison groups [except for 1X(no)- 2X(no)- 3X(no)] as shown in Table 1. We found, these differentially methylated genes were enriched in three processes including cellular components, molecular function, and biological processes (Fig. 3b).

As methylation patterns were sensitive to ploidy changes, and promoter-methylated genes constitute largest proportion among DNA methylated genes. Functional classification was only done to those genes that were differentially methylated in promoter regions (Fig. 3a). GO enrichment analysis revealed most differentially methylated genes (72.75%) belong to “molecular function” domain, followed by cellular component, and biological process domains. The genes enriched in molecular function were characterized with sub-GO terms “binding” and “catalytic activity”. Among all the genes that were highly methylated in promoter regions, the largest proportion belonged to no-up group, while the lowest proportion belonged to down-down and up-up groups, when ploidy changes from 1X, 2X, and 3X (Fig. 3b).

### Correlation of DNA methylation in promoter regions and expression of genes

Using log2 FC ≥1, *P* < 0.05 as cut off, we analyzed the correlation between gene expression and the level of DNA methylation (Fig. 3c). As the results indicated, the level of the DNA methylation is negatively correlated with gene expression within each ploidy level, suggesting that ploidy level did not affect the repressive role of this epigenetic mark on gene expression.

Although, from above results it has been demonstrated that DNA methylation level was found highest (76.39%) in promoter regions, and was unchanged in all three ploidy materials (Table 1). In order to analyze the effect of the methylated regions on the expression of adjacent genes. We examined whether DNA methylation in the promoter regions of different ploidy materials had an impact on gene expression by combining the methylation reads of promoter regions with mRNA expression data. The reads that showed differential variation in methylome map among different ploidy were statistically analyzed. In brief, we classified the methylation variation in promoter and gene expression among haploid, diploid, and triploid seedlings into nine categories; no-down-down, no-down-no, no-down-up, no-no-down, no-no-up, no-no-no, no-up-no, no-up-down, and no-up-up.

A total of 1089 genes were found in mRNA expression profile among different ploidy materials (Fig. 3d). Among them, 672 genes showed changes in gene expression under different ploidy materials that accounted for 61.71% of expressed genes. While, promoter methylation data revealed a total of 27,128 genes, out of them 22.39% (6074 genes) showed changes in methylation of promoter region under different ploidy level. Currently, we analyzed 672 genes that showed differential mRNA expression profiles under different ploidy. We found group [1X(no)- 2X(down)- 3X(down)] showed highest variation in expression of 155 (23.07%) genes, while [1X(no)- 2X(down)- 3X(no)] showed the least changes in expression of 21 (3.13%) genes. While methylome analysis revealed highest methylation in group [1X(no)- 2X(no)- 3X(down)] that contained 2134 (35.13%) genes while, lowest methylation was found in group [1X(no)- 2X(down)- 3X(down)] contained 42 (0.69%) methylated genes. Methylome analysis of 6074 genes revealed that relative proportions of gene expression and methylation level in promoter region of nine comparison group was different. For example, group [1X(no)- 2X(no)- 3X(no)] accounted for 38.29% in terms of gene expression but accounted 77.61% in methylation of genes in promoter region. These results are consistent and showed existence of a negative correlation between DNA methylation and gene expression under the different ploidy level (Fig. 3e).

### Effects of DNA methylation levels in gene expression of promoter regions

**Fig. 4:**
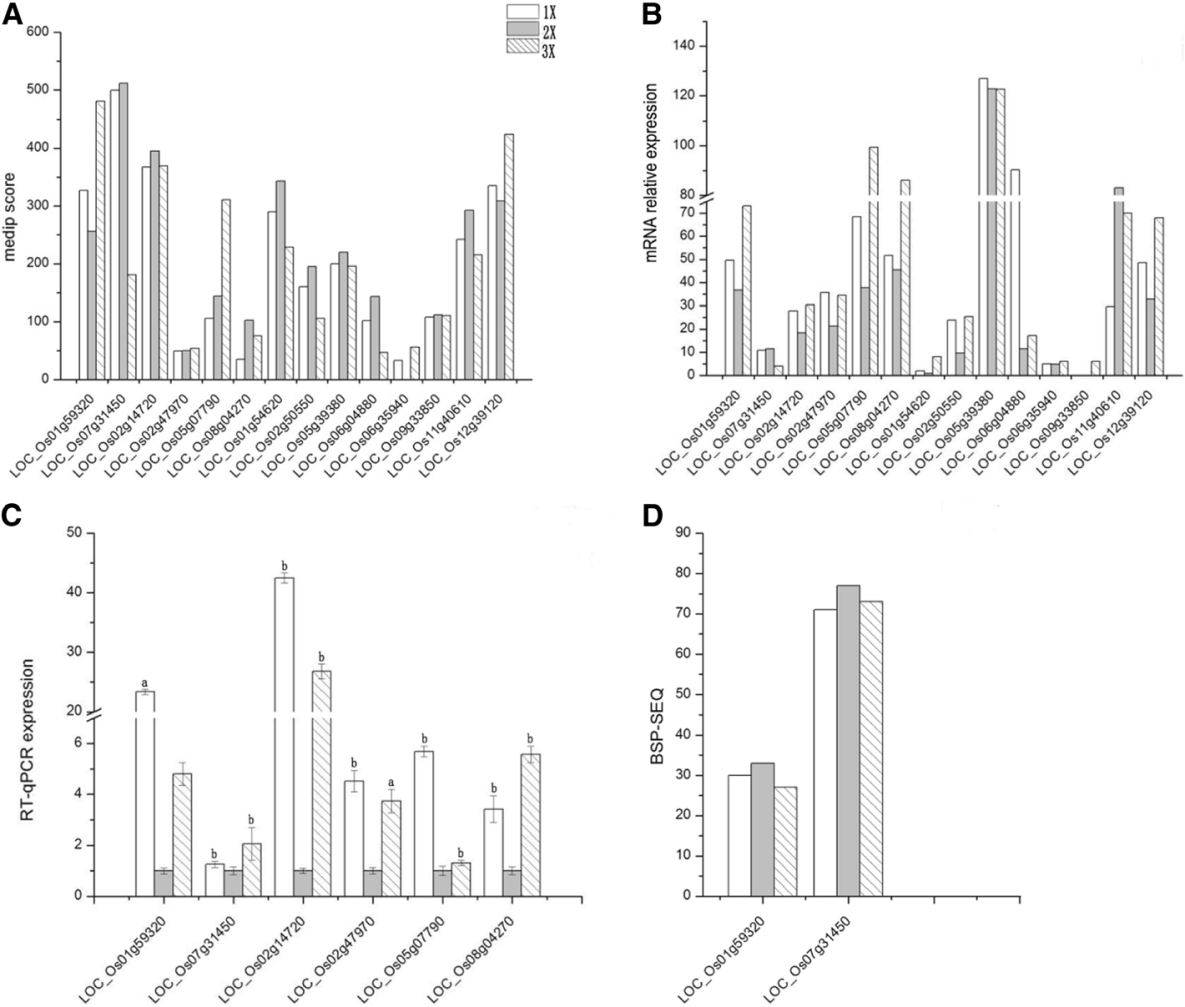
**a** MeDIP score (number of extended methylated reads per Kb in the genome) of 14 genes among haploid, diploid, and triploid seedling. **b** Differential mRNA expression of 14 genes among haploid, diploid, and triploid seedling. **c** Relative expression of six genes among haploid, diploid and triploid seedling. Where, a and b represent significant differences at (*P-*value by Fisher’s exact test) 0.05 > a > 0.01 and b < 0.01. **d** Comparison of the numbers of methylated cytosines in promoter regions of two genes (LOC_Os01g59320 and LOC_Os07g31450) among haploid, diploid, and triploid rice plants obtained though BSP- sequencing

In order to further confirm this relationship, we compared mRNA expression profile with promoter methylation data to further assess whether promoter methylation of a single gene and expression of that gene is affected by ploidy level (Fig. 4a).

We used MeDIP-Seq data to investigate whole genome DNA methylation among three different ploidy (haploid, diploid, and triploid) seedlings (Fig. 4a). Results revealed relative DNA methylation level across different genome was found in following order; diploid > triploid > haploid. We selected 14 genes for mRNA expression showing different methylation among haploid, diploid, and triploid seedlings and examined them in detail based upon single gene expression (Fig. 4b). Results exposed that levels of methylation in promoter regions differed with respect to different ploidy levels (Fig. 4a) and also for their expression (Fig. 4b). The mRNA transcription data for most of these genes showed an opposite trend to MeDIP-Seq data among three ploidy materials. Howbeit, these findings were consistent with the previous observation of negative correlation between DNA methylation levels in promoter regions and their gene expression. We also explored whether presence of smRNA distribution and their expression spectrum was predicted according to rice database in our previous studies [32]. Results of that previous study reveals absence of distribution and regulation of smRNA in these genes. Hence, we hypothesize that variation in gene expression was induced by the changes in DNA methylation present in the promoter regions.

Eight genes were chosen based on above known mRNA expression data (Fig. 4b), and their expression was further verified by quantitative PCR with gene-specific primers (Fig. 4c), We found that seven out of eight genes showed consistent expression trend as with the measured data of mRNA profile selected genes of haploid, diploid, and triploid seedlings. Current results of Q-PCR were (87.5%) consistent with that of mRNA expression data.

We chose LOC_Os01g59320 and LOC_Os07g31450 that showed consistent and inconsistent mRNA expression data, respectively with our known Q-PCR expression profile. BS-Seq analysis were performed further to determine distribution of DNA methylation in the promoter regions. After treatment with bisulfite, the genomic DNA was amplified by methylation-specific PCR. A minimum of 15 positive clones were sequenced to ensure the accuracy of BS-Seq data. The online software Cytosine methylation analysis tool for everyone (CyMATE) (http://www.cymate.org/) was used to analyze the cytosine methylation in 15 BS-Seq clones (Additional file 1: Figure S1). The results showed that the number of methylated cytosine (mCs) sites in the promoter region of the two genes was highest in diploid than triploid and haploid plants (Fig. 4d and Table 2).

**Table 2:** Numbers of cytosine and methylation rates in the promoter regions of two genes in haploid, diploid, and triploid seedling

Gene name	Material	CG	CHG	CHH	C total	Sequence length	Methylation level
C Total		11	9	35	55	369 bp	
LOC_Os01g59320 m-C number	N	10 (90.91%)	5 (55.56%)	15 (42.86%)	30	369 bp	54.55%
	2 N	11 (100%)	3 (33.33%)	19 (54.29%)	33	369 bp	60%
	3 N	11 (100%)	5 (55.56%)	11 (31.43%)	27	369 bp	49.09%
C Total		49	120	317	486	2000 bp	
LOC_ Os07g31450 m-C number	N	34 (69.39%)	8 (6.67%)	29 (9.15%)	71	2000 bp	14.61%
	2 N	34 (69.39%)	12 (10%)	31 (9.78%)	77	2000 bp	15.84%
	3 N	34 (69.39%)	9 (7.5%)	30 (9.46%)	73	2000 bp	15.02%

**Fig. 5:**
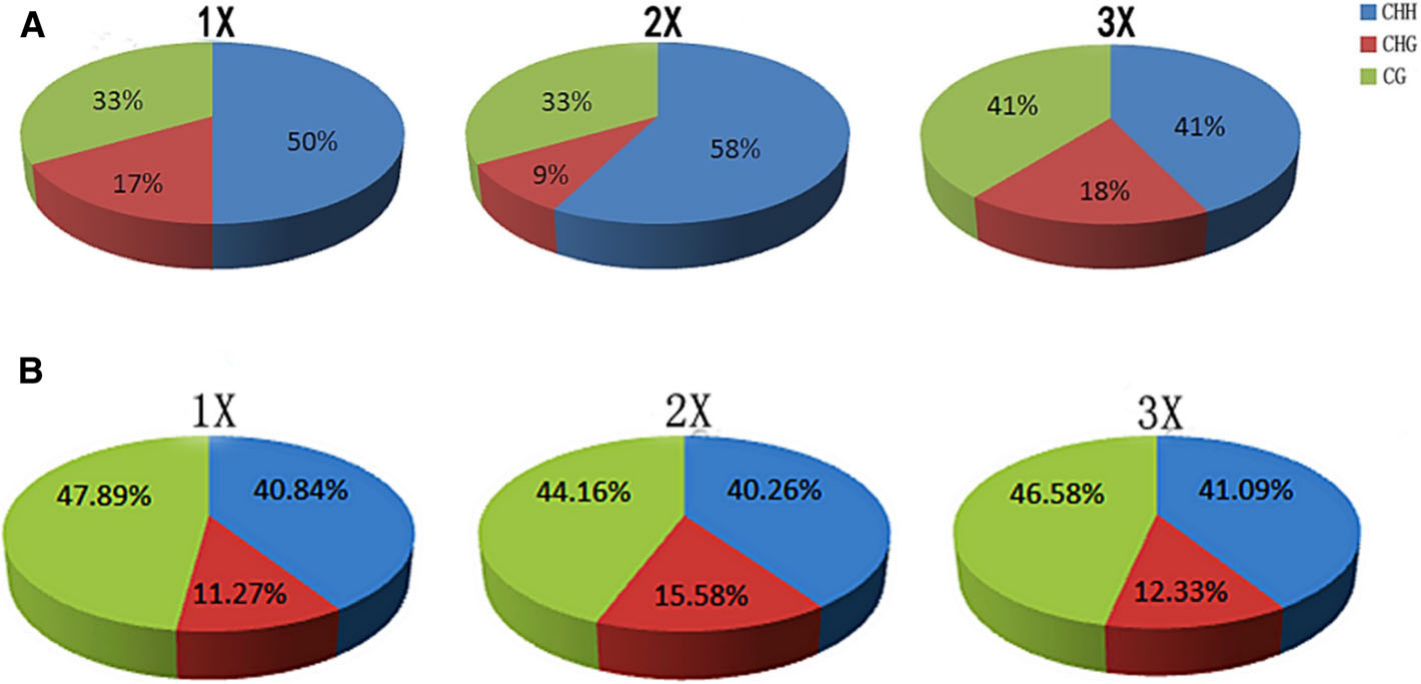
**a** The relative fraction of methylcytosines identified in three (CG, CHG, and CHH) sequence contexts in the promoter region of gene (LOC_Os01g59320) among haploid, diploid, and triploid seedling. Blue, red and green parts of pie graphs are representing fraction of CHH, CHG and CG sequence context respectively. **b** The relative fraction of methylcytosines identified in three (CG, CHG, and CHH) sequence contexts in the promoter region of gene (LOC_Os07g31450) among haploid, diploid, and triploid seedling. Blue, red and green parts of pie graphs are representing fraction of CHH, CHG and CG sequence context respectively

Sequencing of the 369 bp of promoter region of gene LOC_Os01g59320 revealed that there were total 55 cytosines, including 11 in the CG, 9 in CHG, and 35 in CHH sequence context. Judging from the occurrence of the methylation modification rate of CG sequence context ranged from 90.91–100%, and showed absence of significant differences among different ploidy materials. The highest modification was mainly reflected in CHH sequence context among different ploidy materials that accounted highest number of methylated cytosines. Among three ploidy levels cytosine methylation modification pattern was highest in CHH than CG, and followed by CHG sequence context (Fig. 5a and Table 2). Overall, the methylation rate of cytosines in promoter region was found highest in the diploids (33 mCs) comprising highest proportion (54.29%) from CHH and lowest proportion (33.33%) from CHG sequence context.

The promoter region of LOC_Os07g31450 contained 2000 bp, which is comprised of 486 cytosines e.g. 49 in CG, 120 in CHG, and 317 in CHH sequence context. In judging from the occurrence of the methylation rate of CG context was the highest, as well as the number of cytosines is 34 (69.39%), that is same among different ploidy levels. Cytosine methylation pattern among different ploidy levels showed highest number of mCs in CG than CHH and then followed by CHG sequence context (Fig. 5b and Table 2). The CHH (9.78%), CHG (10%) sequence contexts are highest in diploids as compared with the haploids and triploids; therefore, the methylation rate of cytosines (77 mCs) that accounts for 15.84% of total cytosines found in promoter region was highest in diploids.

## Discussion

### Distribution of methylation in CG, CHG, and CHH sequence contexts among promoter region of haploid, diploid and triploid

In current study, methylation rates in promoter regions of LOC_Os01g59320 and LOC_Os07g31450 among different ploidy (haploid, diploid and triploid) material were studied. We found, methylation rates of two selected genes were found highest in diploids than haploid and triploid in following order (*N* < 2 *N*> 3 N). In addition, the pattern of cytosine methylation in three (CG, CHG, and CHH) sequence contexts was analyzed. Among these, methylation in CG sequence context was found highest (mCs rate ranged from 69.38–100%) but did not differ among all three ploidy. Whereas, the methylation rates of the CHG and CHH contexts varied for both genes with different ploidy. For example, the methylation rate of the CHG sequence context of LOC_Os01g59320 among diploids was lowest than in haploids and triploids (1 *N* > 2 *N* < 3 N). In LOC_Os07g31450, CHG sequence context methylation rate was highest in the diploids than in haploids and triploids (1 *N*< 2 N> 3 N), and the methylation rates in CHH sequence context were in following order 1 N< 2 N> 3 N. Previous study [49] established that CHG and CHH sequence context methylation levels in TEs of tetraploid rice were generally elevated. Here, our study found that methylation rate in CG sequence context of promoter regions was highest, and not affected by different ploidy level. Our results are suggesting firstly, demonstrated that the high CG methylation did reduce gene expression change suggesting that DNA methylation exert repressive function and ensure genome stability during WGD. Secondly, ploidy changes in promoter region had a large impact on methylation of CHH and CHG sequence context, especially an obvious effect on CHH sequence contexts. We suspect it could be due to higher methylation rate (317, 65.23%) in CHH sequence context which is much higher than that of CHG sequence context (120, 24.69%). Likewise, stability of CHH methylation was also lower than that of CHG sequence context. Our results validate the findings of Feng et al [1] that reported the CHH sequence context methylation is more widely distributed in rice genome. It is known that decrease in METI and (DDMI) are necessary to maintain the methylation in CG sequence context, while CMT3 mainly maintains cytosine methylation in CHG and CHH sequence context [54, 56]. Cheng et al. [57] demonstrated that *oscmt3a* could decrease CHG sequence context methylation significantly and thereby changes the expression of some genes. Therefore, we suspect that ploidy changes regulate the enzyme activities of METI, DDMI, and CMT3 by changing the specific (or preferred) methylation patterns in CG/CHG/CHH sequence contexts, which in turn regulates the expression levels some genes. Yet verification of this hypothesis still needs further experiments.

### Relationship between DNA methylation and gene expression in haploid, diploid, and triploid

The intergenic regions of genome are highly methylated that generally contains TEs and repeated sequences, but methylation of promoter and intragenic regions has also an important influence on gene expression. Numerous studies have shown that DNA methylation in these two regions can also affect transcription [21, 58]. There are three hypotheses concerning the effects of methylation on mRNA transcription in promoter and intragenic regions of plant genes; (1) methylation in the promoter region inhibits transcription, therefore directly affects gene expression, while the impact of methylation in the intragenic region on transcription is negligible [59]. (2) Methylation in the promoter region does not affect transcription, but methylation in the intragenic region result into down-regulation of gene expression [60] (3) While third hypothesis explains the role both 1 and 2 scenarios have an impact on transcription and gene expression [21, 58].

We used DGE to analyze the average transcription levels of methylated genes in different genic regions among different ploidy level. Our results presented that impact of methylation on gene expression varied with in different gene regions (Additional file 1: Fig. S2). Our findings embrace the above three hypotheses: (1) The average transcription level of methylated genes was highest in the diploid, indicating that methylation in intragenic region of diploid had impact on gene expression, that are consistent with first hypothesis. (2) In triploids, the average transcription level of methylated genes is only higher in promoter region than that of intragenic region, that are consistent with the second hypothesis. (3) In haploid, average transcription level of genes without methylation, were found highest and the gene expression level was slightly lowered, when methylation occurs in promoter region or the intragenic region. But, when methylation occurs in both intragenic and promoter regions, gene expression level was lowest, which is in consensuses with the third opinion.

Zhang et al. [49] also found increased number of copies in genome of tetraploid rice, but the relative expression of most genes was retained at the same levels as in diploid, showing a dose-compensating effect. Investigators speculated, similar expression levels were due to variation in DNA methylation levels of TEs induced by polyploidy impact to genome that restricted expression of surrounding genes. The maintenance of expression level is conducive to buffer the effect of immediate multiplication of genome as cell environment machinery cannot rapidly increase to process loftier expression.

In generally, we think that DNA methylation is not the only mark affecting gene expression in rice seedlings of different ploidy, based on the unclear relationship between gene transcription and DNA methylation in different gene regions. Epigenetic modifications, including smRNA regulation, histone modification, and environmental variations can modulate gene expression in coordination with DNA methylation. During polyploidization of plants, DNA homogeneity and chromosome rearrangement should also be considered in modification of genome in addition to epigenetic modification, which has an important effect on genome evolution.

## Conclusion

SARII-628 produces twin seedlings of different ploidy and we compared the level of methylation and mRNA expression in three different (CG, CHG, and CHH) sequence contexts of promoter region among haploid (1X), diploid (2X), and triploid (3X). MeDIP-Seq analysis revealed that relative level of DNA methylation among different ploidy was in order of diploid > triploid > haploid. Methylome data, digital gene expression, mRNA expression profile, and Q-PCR found that among all ploidy levels, CG sequence context had highest methylation frequency, indicating that CG methylation sequence context plays a dominant role in maintaining gene silencing during WGD. Change in ploidy reveals supreme changes in methylation frequency of CHH sequence context. Our finding will contribute an understanding towards lower stability of CHH sequence context and educate the effect of promoter region methylation during change in ploidy state in rice.

## Methods

### Plant materials

SARII-628 is a specific rice line of Rice Research Institute, Sichuan Agricultural University that produces twin seedlings of different ploidy, including monoploid (1X), diploid (2X) and triploid (3X). Ploidy determination of root meristematic cells ensured the absence of heterozygotes and chimeras in experimental material. STR markers were used to determine whether there were any significant differences in DNA primary structure (at marker loci) in seedlings of three different ploidy types [50–53].

### Methylated DNA immunoprecipitation sequencing (MeDIP-SEQ)

A Plant Dneasy Mini Kit (Qiagen, USA) was used to extract total genomic DNA from flag leaves of rice plants grown under uniform watering and fertilization environments. Three independent biological repeats were setup to collect DNA samples from each ploidy level. The genomic DNA from each individual replicate was pooled for each ploidy. After measuring the concentration with a UV spectrophotometer, the genomic DNA was sonicated to produce random 200–600 bp fragments. The 4 mg DNA fragments were analyzed for standard methylated DNA immunoprecipitation sequencing (MeDIP) at the Beijing Genomics Institute (BGI) [50].

### Transcripts (mRNA) digital gene expression

Total leaf RNA from 1X, 2X, and 3X plants was extracted using Trizol (Invitrogen) followed by DNase treatment. Gel electrophoresis and ultraviolet spectrophotometry were used to determine the integrity and purity of RNA samples. Three independent DGE libraries were constructed using total RNA isolated from 1X, 2X, and 3X seedlings (following Zhang et al. [50]). Then, RNA was sequenced at Beijing Genomic Institute (BGI) using Illumina Genome Analyzer II, and the results were analyzed by BWA (Burrow Wheeler Aligner) software.

### RNA extraction, reverse transcription, and qRT-PCR assays

Total RNA was extracted with Trizol reagent as according to the standard protocol. After purification and reverse transcription, gene-specific fragments were amplified by PCR and detected through electrophoresis. Software Beacon Designer 7.0 was used to design the primers for the target genes across the introns. Primer sequences are shown in Table 3. Gene expression was quantified by qRT-PCR using SYBR Green Master mix (ROX, Roche).

**Table 3:** Primer sequences of PCR

Gene	Forward Primer	Reverse Primer
LOC_Os01g59320	CGCTGGAGTCTGAAGAGATGTC	TCACAAGCCACAGAACAAGG
LOC_Os02g14720	GTTATGGAAGCGATTTGG	GAGATGGCTCAGTTACAGG
LOC_Os02g47970	CGAAGGAGGTATCTATCAGTT	CGCTCAAGAACCAACAGTG
LOC_Os05g07790	TGTGGTTGAAGATGAAGAGG	CCGCAGGAATAGGACGAT
LOC_Os07g31450	GCTGTCCACGAACATACC	TACGACATCTTGGGCATT
LOC_Os08g04270	TATCAGACCAGCCCTCCTGTT	GCCATATGTTGCCATCCTCT
UBC	CCGTTTGTAGAGCCATAATTGCA	AGGTTGCCTGAGTCACAGTTAAGTG
GAPDH	AAGCCAGCATCCTATGATCAGATT	CGTAACCCAGAATACCCTTGAGTTT

### Bisulfite sequencing

The DNA was treated using the standard protocol provided by EpiTect Bisulfite kit (Qiagen). The primers for BS-seq are given in Table 4. DNA fragments were amplified using TaKaRa EX Taq and followed by standard cloning and sequencing protocols.

**Table 4:** Sequencing Primer for Sodium bisulfite sequencing

Gene	Forward Primer	Reverse Primer
LOC_Os01g59320–1	ATAAGTAAATATATAGTATGGGGGTGTTTA	ATTATAAACCCTATTTATATCACACTCTAA
LOC_Os01g59320–2	TCCCACTCACATGAACACG	CGCACAAATCAACATACCG
LOC_Os07g31450–1	GTATTTTATTTAAGTATTTGAAGTTAGTATCGGA	CAAACATAAACGAATAAATTTATTAAACTA
LOC_Os07g31450–2	ATTTATTGAATTTTTTGTATTATCGT	GACGAGATGATTATAGATGAGTATTAG

## Additional file

Additional file 1: **Figure S1.** Graphical output of CyMATE. (**A**) In silico analysis of methylation of LOC_Os01g59320 in haploid with CyMA. (**B**) In silico analysis of methylation of LOC_Os01g59320 in diploid with CyMATE. (**C**) In silico analysis of methylation of LOC_Os01g59320 in triploid with CyMATE. CyMATE filled symbols represent cytosine methylation, while open symbols represent lack of methylation. The sequence context is distinguished by red circles for ^m^CG (Class 1), blue squares for ^m^CHG (Class 2) and green triangles for ^m^CHH (Class 3). **Figure S2.** Average expression level in 1X, 2X, and 3X. Effect of methylation on gene expression in different gene regions haploid, diploid and triploid seedling, where 1X, 2X, and 3X represents haploid, diploid and triploid plants. (ZIP 1892 kb)

## Abbreviations

BS-seq: Bisulphite sequencing
CyMATE: Cytosine methylation analysis tool for everyone
MeDIP-seq: Methylated DNA immunoprecipitation sequencing
STR: Short tandem repeat
TEs: Transposable elements
WGD: Whole-genome duplication
