# Supplementary figures and images for: Effects of ploidy variation on promoter DNA methylation and gene expression in rice (Oryza sativa L.)
Source: BMC Plant Biol. 2018 Nov 29;18:314. doi: 10.1186/s12870-018-1553-5 (PMC6267922; doi:10.1186/s12870-018-1553-5)

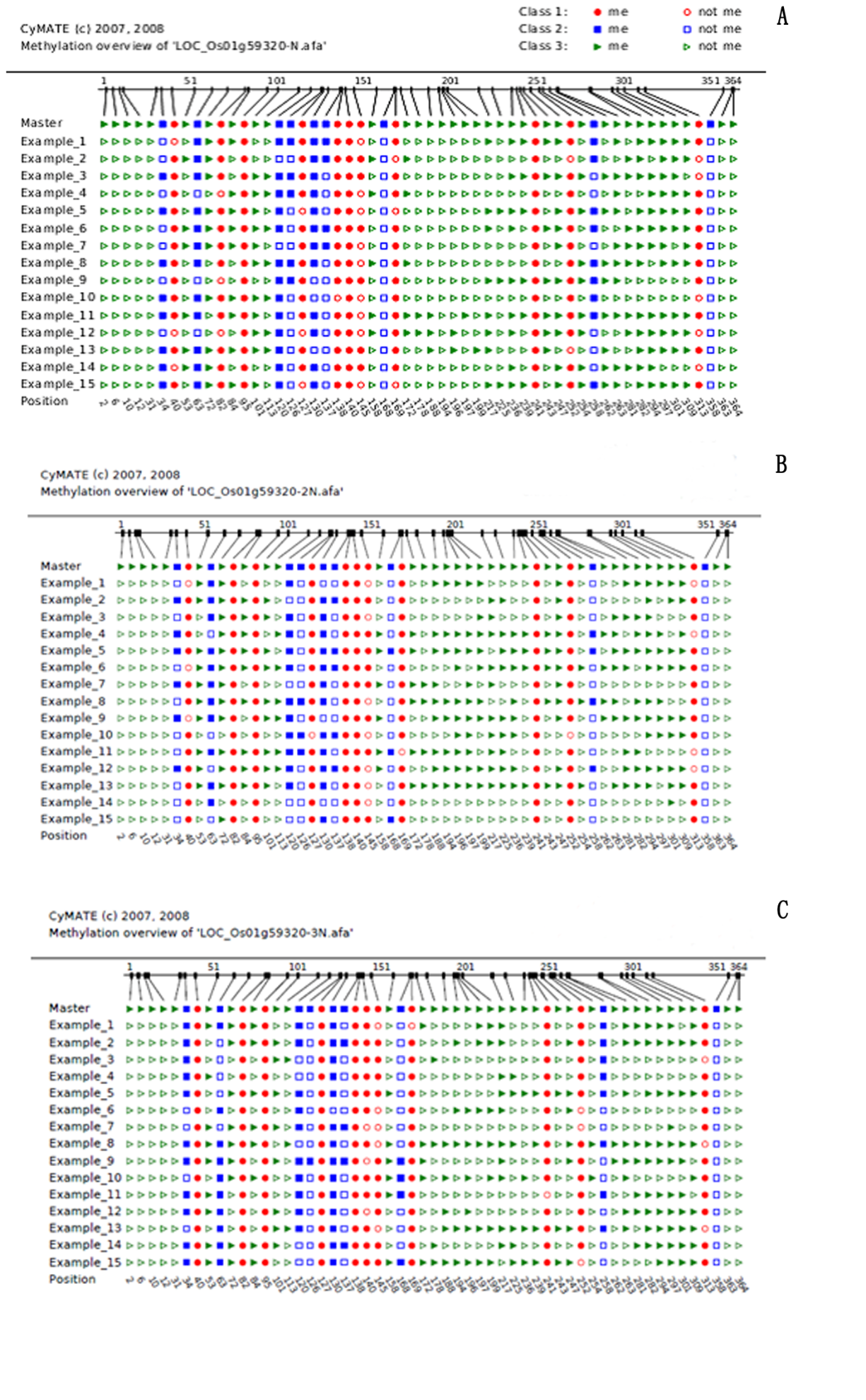


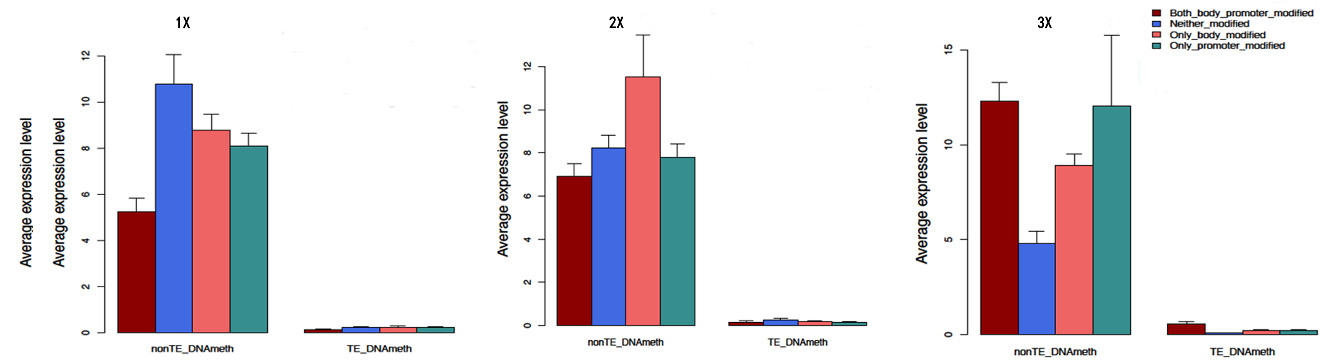

Supplement: Supplementary file 1 — Figure S1. Graphical output of CyMATE. (A) In silico analysis of methylation of LOC_Os01g59320 in haploid with CyMA. (B) In silico analysis of methylation of LOC_Os01g59320 in diploid with CyMATE. (C) In silico analysis of methylation of LOC_Os01g59320 in triploid with CyMATE. CyMATE filled symbols represent cytosine methylation, while open symbols represent lack of methylation. The sequence context is distinguished by red circles for mCG (Class 1), blue squares for mCHG (Class 2) and green triangles for mCHH (Class 3). Figure S2. Average expression level in 1X, 2X, and 3X. Effect of methylation on gene expression in different gene regions haploid, diploid and triploid seedling, where 1X, 2X, and 3X represents haploid, diploid and triploid plants. (ZIP 1892 kb) [file 12870_2018_1553_MOESM1_ESM.zip › Suplementry Figures.docx]
